# Extraocular Sebaceous Carcinoma Presenting As a Nodule over the Zygoma

**DOI:** 10.7759/cureus.6858

**Published:** 2020-02-03

**Authors:** Shashank Kraleti, Swapna Manyam, Kellen Dawson

**Affiliations:** 1 Family and Preventive Medicine, University of Arkansas for Medical Sciences, Little Rock, USA; 2 Pathology, University of Arkansas for Medical Sciences, Little Rock, USA

**Keywords:** sebaceous carcinoma, extraocular variant, skin nodule

## Abstract

We describe a case of sebaceous carcinoma (SC) in a 75-year-old man who presented with a rapidly growing nodule on the left cheek for four weeks.

A 75-year-old man presented with a crusted non-tender nodule on the left cheek that had been present for six months. The nodule showed rapid growth in the four weeks before the visit. Shave biopsy of the lesion was reported as SC.

SC is a rare but aggressive type of skin cancer that can develop from any sebaceous gland in the body. However, it mostly occurs in the eyelids. In this case, SC developed on the left cheek of the patient, which is an atypical presentation. Extraocular variant of SC has a greater potential to metastasize and has a lower survival rate compared to ocular variant. Early and accurate diagnosis followed by a wide excision surgery or Mohs micrographic surgery carries a favorable prognosis.

Early detection of extraocular variant of SC may be difficult. It is imperative for primary care providers to order a histopathology examination to investigate a rapidly growing mass in the head and neck region where there are numerous sebaceous glands.

## Introduction

Sebaceous carcinoma (SC) is a rare yet aggressive cutaneous neoplasm that develops from the adnexal epithelium of the sebaceous glands [1]. SC most commonly occurs around the eyes. Only 25% of reported cases of SC occur at extraocular sites [2]. More than 70% of extraocular variants of SC occur in the head and neck regions where sebaceous glands are most commonly located [3]. Extraocular variants are aggressive and metastasize more rapidly [4]. Both clinical and histopathological differences exist between ocular and extraocular SCs [5]. A recent study by Tripathi et al. suggests that there has been an increased incidence of SC among men and white patients [4]. We report a case of a rapidly growing SC on the left cheek in an elderly white male.

## Case presentation

**Figure 1:**
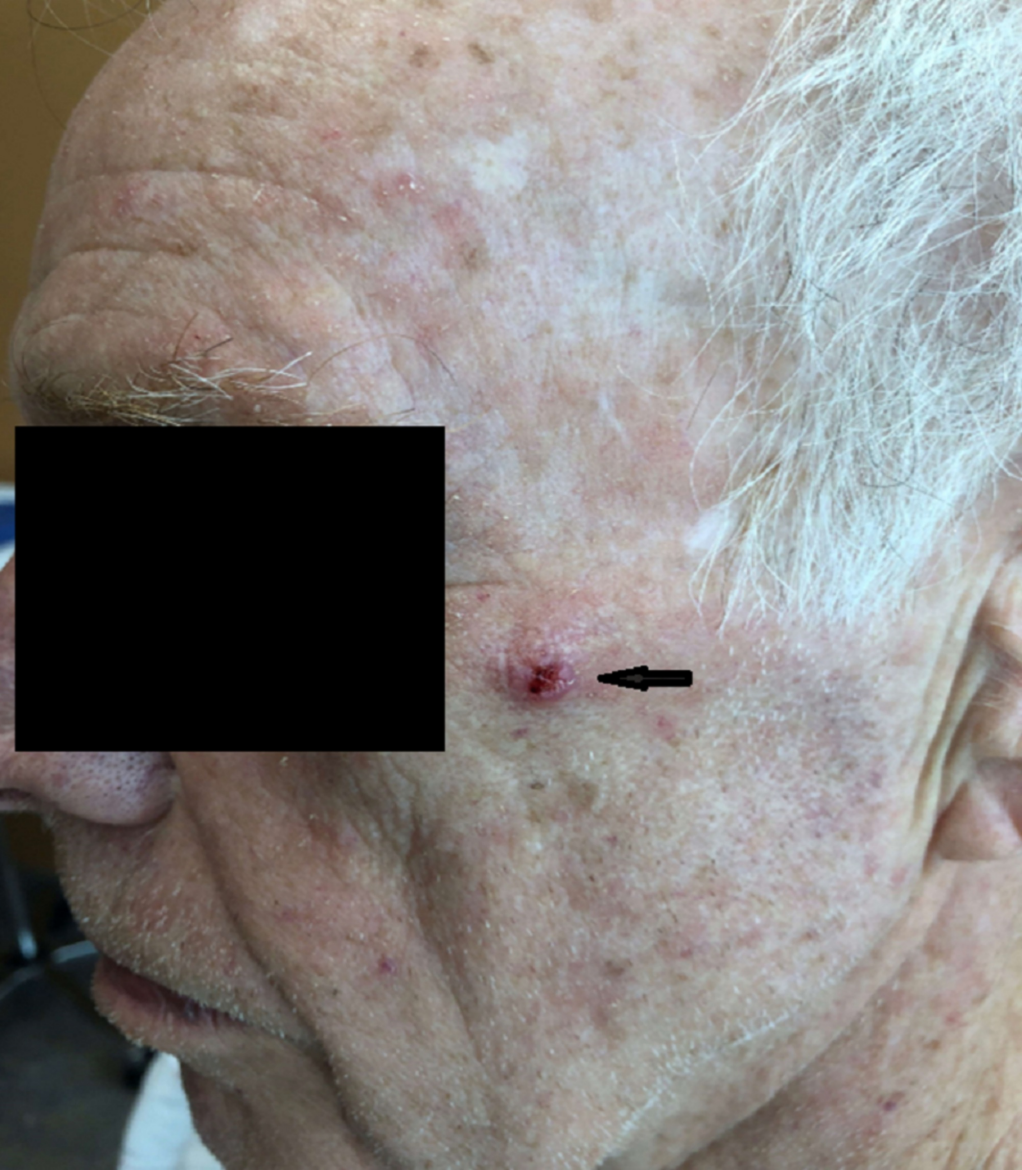
Photograph showing sebaceous carcinoma nodule on the left cheek of the patient.

A 75-year-old right-handed man who was an established patient came to the clinic with a rapidly growing papule overlying his left zygoma. The nodule looked crusted with a central keratin core which had been present for six months but had been growing rapidly for four weeks before presentation to the clinic (Figure 1).

The patient denied any pain or trauma at the location. He had a past medical history of squamous cell carcinoma (SCC) in situ on his nose that had been treated with liquid nitrogen. He also had a history of invasive SCC of left temple that was treated by shave excision without indications of margins. There was no evidence of recurrence of SCC since it had been treated. The patient had a previous history of giant cell tumor of the distal phalanx of the left thumb two years prior to this presentation which was treated by disarticulation at the proximal phalanx of the involved digit.

Otherwise, the past medical history, and social and family history were not significant. On review of systems, it was established that the patient had no fever, chills, cough, or any oral lesions. His medications included aspirin, pravastatin, zolpidem, calcium carbonate, and folic acid. On skin examination, the scalp and forehead had ill-defined erythematous macules with overlying adherent scales. The remainder of the physical examination was unremarkable. The condition was assessed to be a neoplasm of uncertain behavior and was suspected to be a keratoacanthoma type SCC. A shave biopsy was performed. During the same visit, five lesions consistent with actinic keratosis were destroyed using liquid nitrogen cryotherapy.

The final diagnosis of lesion overlying the left zygoma by shave biopsy indicated SC. The patient was informed about the diagnosis and was referred to Dermatology-Oncology.

**Figure 2:**
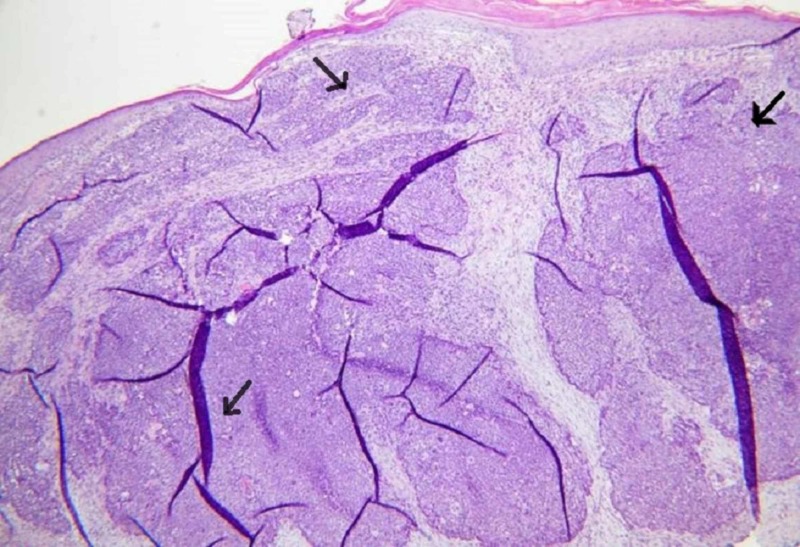
Histological sections reveal skin with unremarkable epidermis and a dermal-based proliferation of sebaceous lobules.

Mohs micrographic surgery of the SC was performed, and the residual tumor was debulked. The surgical specimen showed skin with unremarkable epidermis. There was proliferation of sebaceous lobules in the dermis, which showed basaloid keratinocytes at the periphery and sebaceous differentiation in their centers (Figure 2).

**Figure 3:**
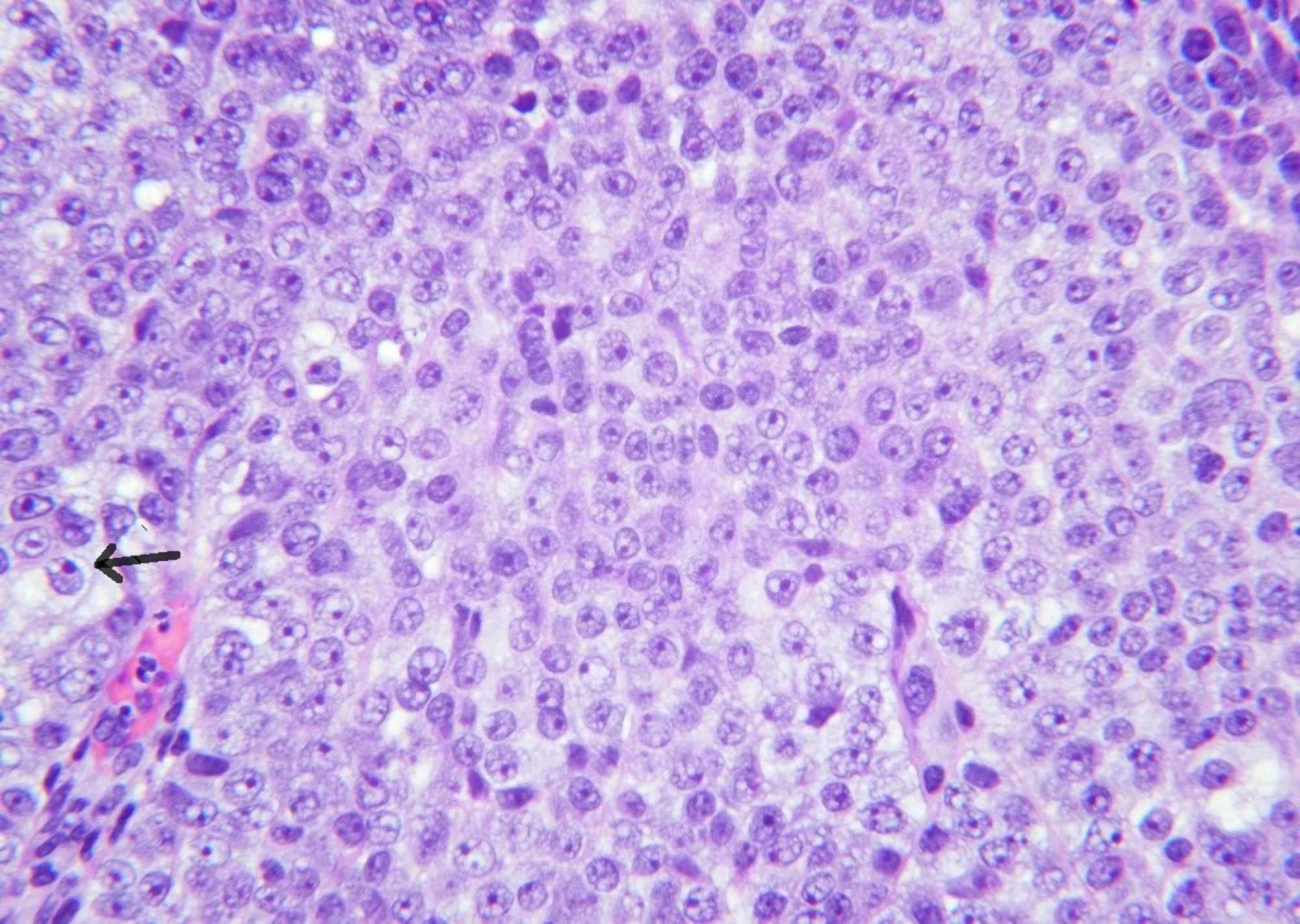
The epithelioid cells display prominent nucleoli and nuclear atypia (40x).

The epithelioid cells display prominent nucleoli and nuclear atypia (Figure 3).

Neoplastic cells showed mature sebaceous differentiation. Overall, architectural growth was minimally infiltrative. On immunohistochemistry, the tumor cells expressed nuclear factor XIIIa, anti-epithelial membrane antigen (EMA), and tumor protein 63 (p63), and were negative for cytokeratin 20 (CK20) and neurofilament.

## Discussion

SC is a very rare type of cutaneous neoplasm that comprises <1% of all skin cancers [1]. However, its incidence has been increasing recently and is much more predominant among elderly white males. Recent increase in incidence can be partially attributed to a better diagnostic capability, but more studies are needed to ascertain the cause. Even though SC occurs predominantly periorbitally, extraocular variants form 25% of all SCs. Additionally, extraocular SC and black race have worse prognoses [4]. From the previous literature, it has been observed that extraocular SC has significant clinical and histopathological differences from ocular SC. Frequent ulceration is observed in extraocular variants, whereas more pagetoid spread of tumor cells in epidermis is seen in ocular variants [5]. In our patient, however, ulceration of the lesion had not been observed. The majority of extraocular SCs are moderately differentiated, while very few may be well or poorly differentiated. Even though the previous literature suggests that extraocular SCs predominantly present as squamoid growth pattern, the histopathological report of our case revealed a basaloid growth pattern. Immunohistochemical findings showed that the tumor cells were positive for EMA which differentiates it from basal cell carcinoma [6]. Histological features that are associated with a poor prognosis and fatal outcome include multicentric origination, poor sebaceous differentiation, intraepithelial pagetoid distortion of the overlying epithelium, and a highly infiltrative growth [7].

Another typical histopathological feature linked to extraocular SC includes having a cutaneous horn and rippled effect [8]. According to Jhuang et al., well-differentiated SC with an overlying cutaneous horn might be a marker of mismatch repair protein deficiency as seen in Muir-Torre syndrome (MTS, a rare autosomal dominant genetic trait characterized by at least one SC tumor and one internal/visceral malignancy) [8]. The most frequent cancers associated with SC are colorectal and genitourinary cancers. The patient in our case presented with a hyperkeratotic papule which necessitated a colonoscopy to rule out the probability of MTS. MTS is related to germline mutations in the MutS Homolog 2 (MSH2) and MutL Homolog 1 (MLH1) genes found on chromosomes 3p and 2p, respectively [9].

The primary treatment of SC is wide excision with a safety margin of 5 to 6 mm with either frozen section or permanent section control or a Mohs micrographic surgery. If metastasis is observed, postoperative care includes radiotherapy and/or chemotherapy. Mohs micrographic surgery was done on our patient, and the outcome was satisfactory without any complications.

## Conclusions

SC is a rare type of skin malignancy accounting for <1% of all skin cancers. It is, however, very aggressive. Prompt and accurate diagnosis of any rapidly growing skin mass is warranted to prevent morbidity and mortality. SC can arise in ocular and extraocular locations and often exhibits different histological growth patterns and diverse clinical presentations. These features may often delay the diagnosis for months to years. Any rapidly growing skin mass, either ocular or extraocular, should raise the suspicion of SC, and an immediate shave biopsy of the involved skin is essential to ensure timely diagnosis. Management options should be considered based on the histological features as well as the extent of spread of the tumor for a favorable prognosis.
